# Evaluation of the durable effects of corrective exercises on the postural alignment and stability in hyperkyphotic elderly with a history of falls

**DOI:** 10.1186/s12877-022-03210-4

**Published:** 2022-06-30

**Authors:** Parisa Sedaghati, Somayeh Ahmadabadi, Maryam Goudarzian

**Affiliations:** 1 Department of Sports Injuries and Corrective Exercise, Faculty of Physical Education and Sport Sciences, University of Guilan, Rasht, Iran; 2 Department of Physical Education and Sports Sciences, Farhangian University, Tehran, Iran; 3 Iranian Research Center On Healthy Aging, Sabzevar University of Medical Sciences, Sabzevar, Iran

**Keywords:** Balance, Posture, Elderly, Motor function

## Abstract

**Background:**

This study aimed to investigate the durability of the effects of corrective exercises on the postural alignment and stability of hyperkyphotic elderly with a history of falls. Balance disturbance and recurrent falls are directly related to changes in the alignment of physical posture and function of the elderly, and effective methods with durable effects on improving the postural stability of the elderly have always been under the attention of researchers.

**Methods:**

This study was a randomized clinical trial, and the statistical population included the elderly referred to neurology clinics. According to the research inclusion criteria (hyperkyphotic elderly with a history of falls during the last six months), 30 elderly aged 60 to 75 years old were purposefully selected and randomly divided into two groups of corrective exercises and control. The experimental group performed an exercise program based on the Alexander posture correction technique. Both groups were evaluated with forward head angle, kyphosis, the timed up and go test, postural stability, and fear of falling tests in three phases. Data analysis was conducted using SPSS 21 software and the MANCOVA test and repeated-measures analysis of variance.

**Results:**

Data analysis showed significant improvements in the variables of forward head (*p* = 0.007), kyphosis(*p* = .001), balance(*p* = 0.002), postural stability(*p* = 0.001), and fear of falling(*p* = 0.001) in the experimental group. Post-test comparisons between the experimental and control groups showed significant differences in all variables except for kyphosis(*p* > .05), and follow-up analysis also revealed significant differences in all variables, except for the variables of forward head and kyphosis(*p* > .05).

**Conclusions:**

Although the effects of corrective exercises in the elderly decreased regarding spinal alignment after three months, more lasting effects were seen in functional balance, postural stability, and fear of falling, suggesting this strategy as a stimulus for more mobility and a lower risk of falling in the elderly. Therefore, when using corrective exercises, it is possible to apply these exercises for a longer period of time to achieve more durable outcomes, especially regarding spinal alignment.

**Trial registration:**

This research was registered in the Iranian Registry of Clinical Trials (IRCT2016081529373N1, Date of registration: 19/04/2017).

## Background

Decreased balance and postural control in the elderly, which occurs following either several neurological disorders or the aging process, cause consequences such as falls. Falling may lead to a variety of problems physical, psychological, and social disabilities, as well as reduced functionality and independence in daily life activities, and finally, death. The prevalence of falling is about 30% in the population over 65 years old, and on the other hand, decreased postural control is influenced by inactivity [3, 24].

In the elderly, falling causes severe consequences such as severe fractures, especially in the bones of the neck, wrist, and pelvis, as well as soft tissue injuries [8]. Also, the results of studies have shown that half of the elderly with a history of falls will develop mobility restrictions [2]. It has been suggested that the practice of physical and sporting activities efficiently counteracts these age-related disorders, reducing the risk of falling significantly [24].

The process of aging is associated with unwanted structural and functional changes, which are particularly accumulated with age. These unsatisfactory changes hinder the performance of motor skills and reduce the individual’s compliance with the environment. In addition, aging changes the natural alignment of the posture, with the curved posture becoming more common in the elderly [5].

These postural changes generally include a forward head, round shoulders, enhanced chest kyphosis, decreased lumbar lordosis, curved knees and thighs, and short stature [12]. Research indicates a prevalence of 20–40% for hyperkyphosis in the elderly [4]. In a longitudinal study on 100 men and women aged 50 years and older, it was reported that the kyphosis angle increased by three degrees per decade. On the other hand, the average angles of chest kyphosis have been reported to be 26 degrees in 20-year-olds, 53 degrees in 60–74-year-olds, and 66 degrees in people over 75 years of age [15].

Some researchers have also noted that the rate of increase in the dorsal arch is accelerated in the seventh decade of life, and this increase in the kyphosis angle is linked with decreased physical function, balance and postural control disorders, reduced walking speed, reduced functional ability, and decreased ability to perform daily routines [17]. On the other hand, advancing age and decreasing physical activity disrupt many physiological functions, including sensory-motor functions, compromising postural control and increasing postural fluctuations in the elderly, which ultimately elevate the likelihood of falls [23]. Sinaki et al*.*, in a study in 2005, investigated postural control in 12 women (average age of 76.5 years) with kyphosis osteoporosis and balance function in 13 healthy individuals (with a mean age of 71 years) using the force-plate. In a recent study, the changes in the foot’s center of pressure were measured as an indicator of balance function. The results showed that postural control was poorer, and the risk of falls and injuries was higher in these individuals compared to the control group [30].

In a study, Jang et al. in 2015 evaluated chest kyphosis and curved posture in older women, and the results showed that eight weeks of chest correction exercises significantly improved the status of chest kyphosis and curved posture. After eight weeks of the correction exercises, chest kyphosis improved by 3.45 degrees in the normal posture and 3.50 degrees while bending over [14].

In another study conducted by Katzman et al., 12 weeks of flexibility and resistance exercises targeting the upper and lower limbs reduced chest kyphosis by six degrees [16]. In 2009, Grindil et al. investigated the effects of Hatha Yoga performed for 24 months (three days a week) on women with hyperkyphosis and showed that these exercises could improve chest structure and stiffness compatibility, as well as the forward head position (with about 11.63% improvement in the normal position). The Hatha Yoga exercises employed in the recent study consisted of the movements focusing on strengthening the back extensor muscles of the spine and improving the flexibility of the muscles surrounding the shoulders and pelvis. Nevertheless, because these exercises were not specific for amending chest position, they could not significantly improve the kyphosis angle of the dorsal spine [11]. Therefore, there is a need for certain correction exercises to achieve specific amendments in the postural alignment and to correct the position of the spine [27]. The point that has been less addressed in the correction of the forward head posture and kyphosis is the secondary positive effects of these changes on balance and their role in preventing people from falling. Therefore, the correction of posture can be one of the methods to improve balance and postural control in the elderly.

On the other hand, some studies have employed general as well as specialized exercises (i.e. Otago exercises) to improve balance and postural stability in the elderly [19]. In this regard, Shahrbanian et al. (2019), in a study, compared the effects of physical activity and neuromuscular exercises on postural stability and the likelihood of falls in older females. The results showed a significant improvement in postural stability and a reduction in the risk of falls in the exercise group compared to the control group. In addition, it was revealed that neuromuscular exercises had a better effect compared to physical activity on the likelihood of falls in older women [28].

Considering the motor problems and the postural condition of the elderly, each of which exacerbates the other, it is necessary to employ effective methods to not only improve old people’s quality of life and independence but also maintain their health and delay aging-associated changes. There is a research gap in the evaluation of the durability of the corrective exercises aiming to improve posture, functional balance, and postural stability in the elderly. Therefore, in this study, we investigated the durability of the effects of corrective exercises on postural alignment and stability in hyperkyphotic elderly with a history of falls.

## Methods

**Fig. 1:**
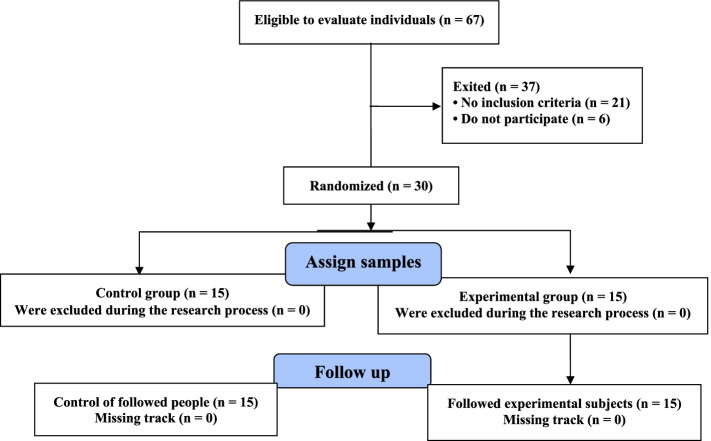
CONSORT flowchart of the allocation of participants to the study groups

This research was a randomized clinical trial with a pre-test & post-test design. The statistical population of this study consisted of elderly men and women referring to the medical centers of Kashan. Regarding the continuous primary outcome variable of the study, an average effect size of 50%, 95% confidence, and the power of 80%, the sample size for pair-comparisons of means and according to a previous study [11] was determined as 28 using G-Power software. The participants were simple randomly (by lottery) divided into the experimental and control groups. In order to match the groups for gender, equal numbers of men and women were assigned to each group. Figure 1 shows the CONSORT flowchart of the study and the process of the allocation of subjects to research groups. Also, Reporting checklist for randomized trials Based on the CONSORT guidelines used for this study, can be found in Appendix S1. All the participants completed and signed informed consent, and all ethical considerations were observed according to the Helsinki Declaration. The research has been approved by the institutional research committee and registered at the Iranian Registry for Clinical Trials and was registered in the Iranian Registry of Clinical Trials (IRCT2016081529373N1, Date of the first registration: 19/04/2017). Also, the study was approved by the ethics committee of Sabzevar University of Medical Sciences, Center for Health Research elderly (the ethics committee reference number IR.MEDSAB.REC. 1398.045).

Inclusion criteria were the followings: forward head with an angle of 46 degrees or wider, kyphosis of 45 degrees or higher, not using aids to walk or perform other daily activities, not having other acute and chronic physical, mental, psychological, and other disorders (e.g., cardiovascular, respiratory, skin problems, osteoarthritis), the ability to regularly participate in training sessions (the maximum number of lost sessions was considered two during the entire study period), having no history of regular exercises, age between 50 to 70 years, the ability to stand for at least one minute and walk 10 m unaided, normal vision (or adjusted vision), ability to follow simple instructions, and an MMSE score of above 24.

Exclusion criteria were a history of depression or other psychological disorders, severe deformities or orthopedic problems of lower limb or trunk joints, history of lower limb joint replacement, history of balance disorders and recurrent positional vertigo, severe pain in lower limb and trunk joints, vestibular diseases, severe visual impairment, unwillingness to continue participation in the study, and not participating in three consecutive training sessions.

The experimental tests were conducted in the three phases of the pre-test, post-test, and three-month follow-up. The primary outcome measures of this study were the evaluation of the forward head angle, kyphosis, the standing up and walking test, the fear of falling test, and postural stability (using the functional accessibility and 180 $^\circ$-turn tests).

### The mental state test (short form)

This test is used to diagnose and screen for dementia and includes the items of spatial and temporal orientation, recent memory, instant memory, reading, writing, repeating, and shape [10].

### The forward head angle

In the present study, the forward head angle was determined by imaging the head at the side-view position and pointing to the landmarks of the ear tragus and the seventh neck vertebra. This method has good reproducibility and has been used in various studies [32].

### Kyphosis

To evaluate the curvature of dorsal kyphosis, a flexible ruler was used and its reliability has been reported to be high [4]. To perform this test, the participants were asked to stand so that the weight is equally distributed on both legs; then, a flexible ruler was placed between the second and twelfth dorsal vertebrae on the subject’s spinous process, so it takes the same shape of the dorsal and lumbar spine arches. The spinous process of the second and twelfth dorsal vertebrae, which were previously marked on the ruler, was marked on the paper. On the shape obtained on the paper, the two points representing the second and twelfth vertebrae were connected with a straight line. The perpendicular line to the arc was then drawn to obtain the kyphosis arc shape. The measurement of the dorsal and lumbar arch angle was repeated thrice for each participant, and the mean value was recorded as the dorsal curvature angle. Finally, the kyphosis angle was calculated using the "Ө = 4arc tan (2 h/l)" formula [14].

### Functional balance (the timed up and go test)

The test was designed by Matthias [21] to assess functional balance and the risk of falling and has high validity and reliability (0.99) [4]. The time in the TUG test encompasses sitting on a chair, getting up, walking three meters, returning, and sitting on the chair again, which is recorded by a chronometer.

### The 180 $^\circ$-turn Test

This test is an indicator to assess postural stability in the elderly. During this test, the individual gets up from the chair and rotates 180 degrees while the hands are placed freely around him/her so that the face is at the opposite point of the test start position. In this test, the number of steps required to complete the maneuver reflects postural stability, and more than four steps in this test indicate limited postural stability and increased risk of falls. With each one-step increase in this test, the risk of falling rises by 22% in subsequent years, and the results of studies have shown a significant difference in the number of steps between people with and without the risk of falling [29]. The reliability of this test has been reported to be 0.828 [9].

### Fear of falling test

The short-form falls efficacy scale-international (short FES-I) was used. The short FES-I is reliable and useful in clinical practice. It is also validated for older adults with cognitive impairment [1, 19]

### The protocol of corrective exercises

The participants of the experimental group performed an exercise program based on Alexander posture correction techniques, one hour per session, three sessions a week for 8 weeks while participants of the control group received general health advice and conventional treatment. The Protocol of Corrective Exercises included the physical training recommended for maintaining and improving the proper posture during daily life activities in the elderly (i.e., Alexander exercises) [7]. The protocol consisted of Alexander exercises, including educating how to maintain the correct posture during daily activities, walking while keeping a straight posture by placing a hypothetical book on the head (forward, backward, and sideways), tandem walking with the straight posture, walking on the toes and heels, and walking with the same and opposite hands and feet (with a straight posture) [26, 31].

### Statistical analysis

For data analysis, SPSS 21 statistical software was used. To evaluate the normality of the data, the Shapiro–Wilk test was utilized. For within-group comparisons, repeated-measures analysis of variance was employed, and between-group comparisons were conducted by the MANCOVA test.

## Results

The demographic characteristics of the participants (means, standard deviations, and frequencies) have been presented in Table 1. The results of the Shapiro–Wilk normality test revealed that the data had normal distribution (*p* > 0.05). Also, data sphericity was confirmed by Mauchly’s statistic, and the results of repeated-measures analysis of variance indicated significant differences in the research variables in the experimental group (*p* < 0.05) while there was no significant difference comparing the research variables between the three study phases in the control group (*p* > 0.05).

**Table 1:** The descriptive characteristics of the Elderly in the Study Groups (*n* = 15)

Variable	Experimental group (M ± SD)			Control group (M ± SD)		
Age (years)	66.00 ± 2.44			65.13 ± 2.74		
Height (cm)	167.5 ± 6.09			163.8 ± 5.28		
Weight (kg)	70.06 ± 5.65			67.66 ± 6.67		
Body mass index (m2 / kg)	24.96 ± 1.60			25.16 ± 1.16		
MMSE	26.07 ± 1.28			25.53 ± 0.99		
**group**	**Experimental (SD ± M)**			**Control (SD ± M)**		
**Variable**	**The first stage**	**The second stage**	**The third stage**	**The first stage**	**The second stage**	**The third stage**
FHD	46.33 ± 4.38	47.33 ± 4.38	47.07 ± 4.66	47.00 ± 3.11	45.73 ± 3.21	46.20 ± 2.80
*P*-value		0.007*			0.090	
Kyphosis	53.60 ± 2.77	52.13 ± 2.94	51.80 ± 3.05	52.93 ± 3.78	52.47 ± 3.75	52.20 ± 3.70
*P*-value		0.001*			0.257	
Functional balance	14.40 ± 1.50	12.93 ± 3.12	13.53 ± 0.99	14.27 ± 1.38	14.07 ± 1.22	14.33 ± 1.17
*P*-value		0.002*			0.681	
Functional reach	22.07 ± 2.28	24.93 ± 3.12	24.53 ± 2.74	21.80 ± 1.56	21.20 ± 2.07	20.73 ± 2.89
*P*-value		0.001*			0.290	
Rotate 180 degrees	4.67 ± 0.90	3.87 ± 0.64	4.13 ± 0.51	4.33 ± 0.72	4.27 ± 0.59	4.40 ± 0.50
*P*-value		0.001*			0.565	
Fear of falling	40.47 ± 3.09	37.53 ± 3.18	38.27 ± 3.73	39.60 ± 3.54	40.33 ± 3.59	40.07 ± 3.43
*P*-value		0.001*			0.157	

^*^ Significance at the level of *P* < 0.05

The pair-comparison of repeatedly measured variables using the Bonferroni post-hoc test (Table 2) showed that in the experimental group and following performing correction exercises, significant improvements were observed at post-test in all variables compared to pre-test (*p* < 0.05). In the experimental group, there were also significant differences in all variables, except for the forward head angle, between the pre-test and the follow-up phase (*p* < 0.05). In the control group, however, no significant differences were observed in any of the variables comparing different phases of the study (*p* > 0.05).

**Table 2:** Pair-comparisons Between Various Study Phases Using the Bonferroni Post-hoc Test

Variable	group	stage first–second	stage second-third	stage first-third
FHD	Experimental	0.048*	0.122	0.155
	Control	0.199	0.451	0.743
Kyphosis	Experimental	0.002*	0.796	0.006*
	Control	0.750	1.000	0.625
Functional balance	Experimental	0.025*	0.171	0.040*
	Control	1.000	1.000	1.000
Functional reach	Experimental	0.005*	0.026*	0.010*
	Control	0.534	1.000	0.614
Rotate 180 degrees	Experimental	0.016*	0.122	0.044*
	Control	1.000	0.493	1.000
Fear of falling	Experimental	0.001*	0.155	0.001*
	Control	0.357	0.786	0.831

^*^ Significance at the level of *P* < 0.05

Table 3 shows the results of between-group comparisons using the MANCOVA test. Considering the pre-test phase as the covariate, there were significant differences between the experimental and control groups in the post-test comparing all the variables except for the kyphosis angle (*p* < 0.05). At the follow-up period, there were also significant differences between the experimental and control groups for all variables except for the forward head and kyphosis angles (*p* < 0.05).

**Table 3:** Between-group Comparisons Based on Multivariate Analysis of Covariance (the Pre-test Phase as the Covariate)

Variable	group	Type III Sum of Squares	Degrees of freedom	Average squares	F	*P*	Eta
FHD	Post-test	34.878	1	34.878	9.824	.005*	.309
	Follow up	14.866	1	14.866	4.012	.058	.154
Kyphosis	Post-test	7.008	1	7.008	3.278	.084	.130
	Follow up	7.001	1	7.001	1.832	.190	.077
Functional balance	Post-test	8.425	1	8.425	7.521	.012*	.255
	Follow up	5.920	1	5.920	6.378	.019*	.225
Functional reach	Post-test	71.575	1	71.575	13.719	.001*	.384
	Follow up	81.527	1	81.527	12.712	.002*	.366
Rotate 180 degrees	Post-test	1.785	1	1.785	5.164	.033*	.190
	Follow up	1.012	1	1.012	7.109	.014*	.244
Fear of falling	Post-test	92.163	1	92.163	42.352	.001*	.658
	Follow up	47.065	1	47.065	23.025	.001*	.511

^*^ Significance at the level of *P* < 0.05

## Discussion

In this study, we aimed to investigate the durability of the effects of eight weeks of correction exercises on spinal alignment, functional balance, and postural stability in hyperkyphotic elderly with a history of falls. The results showed that in the experimental group and under the influence of posture correction exercises based on Alexander techniques, there were significant differences in all research variables between the three phases of the study. In this regard, significant improvements were seen in all the variables in the experimental group between the pre-test and post-test steps. Comparing the pre-test and follow-up periods, there were also significant improvements in all variables in the experimental group, except for the forward head angle. However, in the control group, no significant differences were observed in the variables comparing the three study phases. On the other hand, a comparison between the experimental and control groups revealed significant differences in all variables, except for the kyphosis angle at the post-test and the forward head and kyphosis angles at the follow-up.

Old people generally suffer from motor restrictions due to osteoporosis and spine vertebrae deformities, so they must avoid performing intense exercises, as well as bending and rotating the trunk [18]. Therefore, it is recommended to train these people to obtain and maintain the correct posture and guide them to drop wrong postural habits by performing corrective exercises that are not only simple to do but can also be implemented with minimum facilities and supervision at home by all people, including the elderly. The participants in this study were sedentary elders who had no regular physical activity. Although they were able to move unaided, they were afraid of falling due to a history of occasional, and in some cases, recurrent falls. Fear of falling, in the form of a flawed cycle, causes inactivity, which subsequently leads to reduced motor functions and independence. Therefore, corrective exercises, in addition to training the elderly on how to maintain the correct posture during daily activities, can enhance the level of physical activity, enable these people to walk while keeping the correct posture, and finally improve their functional balance and postural stability. Postural control requires communication and interactions between the nervous and musculoskeletal systems. The components of the musculoskeletal system include a joint range of motion, spinal flexibility, muscle characteristics, and biomechanical relationships between different parts of the body [12]. Physical activity plays an important role in correcting movements, so it is amenable to acquire better motor coordination with surroundings by employing and educating the proper posture.

In fact, this training method, in addition to designating the alignment of the spine during movements, strengthens the deep postural muscles that support this alignment [6]. In addition, the onset of the contraction of central muscles before limb movements triggers the postural prediction response by the central nervous system. Therefore, the exercises that emphasize the stability of core muscles can improve activity prediction, thus reducing balance disorders and central oscillation [20]. In this regard, Kang et al*.* (2015) examined the effects of specialized exercises for the central part of the body on weight stability and distribution in the elderly [22], and their results were consistent with the findings of the present study.

In line with our results, Jang et al*.*, reported that eight weeks of correction exercises with Traband significantly improved the kyphosis angle, balance, and postural stability in older Korean women [13]. Also, in another study, Naderi et al. examined the effect of 12 weeks of kyphosis correction exercises on the physical function and balance of hyperkyphotic elderly men and reported significant improvements in the kyphosis angle and balance in these individuals [21]. In a study, Mohamadtaghi et al. investigated the effects of postural corrective exercises on postural stability in hyperkyphotic elderly women and showed that these exercises not only reduced the kyphosis angle from 50.58 to 48.84 but also improved postural stability in the participants [20]. In another study, Roller et al*.* (2017) assessed the role of modified Pilates exercises in reducing the risk of falls (by modifying the factors involved in falls, i.e., balance, mobility, self-efficacy, and active motion range) in the elderly aged 65 years and older and at the risk of falls compared with the control group. The results showed that selected Pilates exercises reduced the risk of falls and markedly improved static and dynamic balance, motor function, self-efficacy balance, and lower limb range of motion in the elderly [25].

According to our results, spinal alignment, balance function, postural stability, and fear of falling significantly improved at the end of the training period. In addition, although these effects on the motor functions persisted at the follow-up, the effects on postural alignment reversed after three months, indicating the short durability of the effects of eight weeks of correction exercises on the posture and highlighting the need for constant reminders and education to maintain proper body posture in individuals. This is because wrong postural habits during daily activities are institutionalized over time, requiring practicing over and over to maintain the correct posture in each daily activity. These exercises are supposed to gradually lead to better coordination in the musculoskeletal system and therefore improved neuromuscular control. With this strategy, correct habits will replace wrong movement habits and patterns. Therefore, it is suggested to compare the effects and the durability of different types of corrective exercises in future studies and for longer periods (e.g., six months). One of the limitations of this study was the lack of access to more participants to be able to compare the effects of these exercises between men and women and the lack of careful monitoring of the participants’ nutritional conditions and miscellaneous daily physical activities.

## Conclusion

According to the results of the present study, it can be said that corrective exercises based on teaching postural correction and retraining motor activities can improve functional balance and postural stability in the elderly. However, the persistence of the effects of these exercises can be influenced by the duration or type of these exercises, necessitating further studies.

## Data Availability

All data generated or analyzed during this study are included in this published article.
